# A Plea for the Higher Scientific Education of the Individual Members of the Profession

**Published:** 1896-09

**Authors:** Wm. A. Mills

**Affiliations:** Baltimore, Md.


THE
AMERICAN JOURNAL
—OF—
DENTAL SCIENCE.

VOL. XXX. BALTIMORE, SEPTEMBER, 1896. NO. 5.

ARTICLE I.

### A PLEA FOR THE HIGHER SCIENTIFIC EDUCATION OF THE INDIVIDUAL MEMBERS OF THE PROFESSION.

BY WM. A. MILLS, D. D. S., BALTIMORE, MD.

Read before the Union Meeting of the Maryland State Dental Association and the Washington City Dental Society, at Washington, D. C., May 8, 1896.

We have lately considered the subject of dental education from many stand-points, and after much serious reflection, we have been forced to the conclusion, that we stand to-day, whether we are a specialty of medicine or not, in need of more than a superficial knowledge of the science; a necessity which many of us are not very willing to admit, but nevertheless, it is true.

We are not as learned in many things as our title of doctor would infer. If any one doubts this assertion, let the average dental practitioner try to investigate scientifically any of the many phenomena, with which he has to contend, and if he is a conscientious seeker after truth, he will soon find his ability for research, will meet with obstacles which he has no power to surmount.

The reason why so many have failed in this particular, is because we have confined our studies too closely to the *common* diseases of the teeth, met with in our every day practice; and have paid too little attention to the physiologic, pathologic, histologic, etiologic studies, etc., upon which the scientific basis of our profession rests.

Not long ago while seeking information upon the subject of migrated teeth, we asked some of our most prominent practitioners, if the statement made under title of Migrated Teeth, page 412, vol. III, of American System of Dentistry* was true or not? They all answered in the language of the agnostic, "I don't know."

The question has been asked, what have we to do as dental practitioners, with the *fine points* in such cases? We answer: We are called doctors, and if doctors, then we are teachers. Webster says, "Doctor means one who has received the highest degree in a faculty; one who has received a diploma from a university or college, authorizing him to teach; one qualified to teach; one skilled in a profession; to impart as knowledge before unknown," etc.

If this is the correct meaning of our degree, many of us have failed to comprehend its purport; for besides being competent to relieve our patients of many aches and pains, and the substitution of different materials for lost tissues; it is expected of us, intelligently to instruct those who may seek any information, concerning the things which may be directly, or indirectly connected with our specialty; whether it be a practical or theoretical question.

A lawyer may be asked his opinion of a point in law. He can say call again; after I have consulted the authorities, I will let you know. Not so with doctors; they are supposed to be qualified to give an immediate answer, otherwise, their patients or interrogators may think they

*See Dental Cosmos July 1896, page 586.

lack the professional knowledge, they should possess as scientific men.

We often have patients while infected with syphilis, (the primary cause of many of the ills to which the human flesh is heir) come to us to have dental services rendered; is it not our duty to diagnose the disease at once, for the sake of our innocent patients, and our own reputation? How many of us ever think of it, much less recognize it?

Again, in many cases, we are called upon to relieve pains, when the cause of the lesions, lies beyond the confines of the maxillæ, or associated parts; and may require systemic or surgical treatment for its cure. Is it not therefore, very important that we should have sufficient knowledge of the practice of medicine, to enable us to make an intelligent diagnosis? And if not in our province to give the necessary treatment, to recommend them to the physician or surgeon, for their special care and attention? But how many of us have the training to enable us to make a correct diagnosis in such cases?

Dr. B. B. Smith says,* "The dental college course of to-day, includes sufficient of medical science to prepare a man to treat any case (provided) he is capable of diagnosing."

Dr. T. M. Allen says,* "If a dentist has been properly taught all the therapeutics, and materia medica, that is taught in the best dental colleges of to-day, he will know how to treat any case he may encounter, (provided) that he knows how to diagnose."

The word *provided*, in parenthesis, is our own; we use it to emphasize the fact that all proper treatment depends upon one's power to make a correct diagnosis, and these the majority of us do not possess.

Of what use are any weapons of defense, if we do not know how, when, or where to use them to the best advantage?

---

*Dental Cosmos. March 1896, page 251.

Of what use to the dental practitioner are therapeutics, and materia medica, if he cannot make an intelligent diagnosis?

This is the weakest point with many to-day, and for the reason that many of the dental colleges cannot give the proper instructions to make correct differential diagnosticians; because they have not the opportunities, or the means; to give their students the necessary clinical training, which is essential in the study of pathologic conditions.

Another question to which we wish to call attention, is the contempt with which some of our members look upon the journals and text-books, devoted to the elevation of the profession.

Not very long ago, we asked a practitioner (a regular graduate) to what medical or dental journals he subscribed, and no attention was given our query.

We asked again in a louder tone of voice, and he replied, "I don't take any." We said, Doctor, how do you expect to keep up with the rapid advances of the profession? He gazed upon us with contempt, and replied in the words of the classic Greek, "Is there some new thing?" "I read in the *news-papers* some time ago, something about *bugs*, (he meant microbes) is there any thing else?"

We made no reply, for his profound (?) knowledge completely squelched us. To have contended with one so learned, would have been "wasted sweetness on a desert air." Such indifference, or ignorance should find no place in the ranks of the dental profession.

These cases verify our opinion, that all applicants seeking admission to the profession, should possess sufficient literary knowledge to enable them to enter intelligently upon the work, of obtaining special qualifications, that they may become worthy members. Dr. Bond, one of the founders of the dental profession, says in his work on "Dental Medicine." *The Baltimore College of Dental Snrgery, was organized with the desire of teaching Dentistry as*

*a regular branch of medicine, in which relation only, it can be regarded as a scientific pursuit and the practice of it, esteemed a profession.*"

All the original instructors, and supporters of the college were of the same opinion, and so expressed themselves. Notwithstanding such historical facts, it has been publicly asserted that "Dentistry (is) a distinct and independent profession."* "To be a competent dentist, one only needs an experienced eye, and a skilled hand."†

"An educated hand is more to be desired than an educated head,"‡ and the scale of Dental Surgery "rises no higher than the treatment of a felon on a finger."* Such public expressions as these, made by those prominent in the profession, and similar declarations uttered by others; are nothing more than the last expiring echoes, of the teachings of the past.

The time has long since gone, when it was thought, that all things needful for full instruction in the dental colleges, was only a *smattering* of anatomy, physiology, materia medica, chemistry, etc.

This kind of *sufficiency* may have suited our forefathers during the embryonal period of the profession, but like Surgery, and Gynecology, we have since grown to nobler and more scientific proportions; and to-day, it is claimed that the foundation of the Dental profession, (the most complex of all) rests not only upon medicine and its affiliated branches, but also upon many of the fine arts and sciences.

Dr. B. Holly Smith says,* "The dental schools of the present are not those of past years, they teach more of

---

*An address delivered before the Washington City Dental Society, 29th annual meeting.

†Proceedings of the Southern Dental Association, 1882.

‡In debate before the Washington City Dental Society, December 1894.

*The Journal of the American Medical Association, Feb. 29, 1896, page 412.

*Dental Cosmos, March 1896, page 251.

medicine, they go deeper in the sciences." If the dental colleges keep pace with the demands of the profession, their Alumni may not of necessity be graduates of medicine, save only for the honor which may be conferred in the degree M. D.; but if they can read the signs of the times aright, in addition to that taught them by their alma mater, it is requisite, that they continue their studies after graduation, and that they give much time to the study of the many underlying principles of the p rfession, which are not at the present time, given any special place in the college curriculum; one of which we mention, that of Bacteriology, as especially important in view of the fact, that the investigations in this science, have been so marked, that many of the theories laid down as correct, in Physiology, Pathology and Therapeutics, will have to be revised and rewritten. The degree M. D., or D. D. S., at the terminus of one's name amounts to nothing, as being any evidence of ability, or knowledge: we must be scholars, otherwise our titles are but empty honors. Dr. Thackston* says, "No true professional man completes his education 'till disability or death arrests his researches, and forever ends his progress. The dentist who is not daily learning is daily decaying." "He must make progress or he will lose ground. 'Always a scholar' must be the motto of every professional man."

Again he says in addressing a class of graduates, "We might tell you that in severing your connection with the college you should become none the less diligent students; that your application and research so far from abatement should now be more earnest—should take a wider range and broader scope; that you should not only keep abreast with the progress and improvements in your department, but lend your assistance in exploring the yet untrodden fields, and in solving the yet hidden problems of Dental Science."

We are in full accord with Dr. Thackston—we cannot explore the "untrodden fields," etc., as he suggests, except by diligent study of *all things* pertaining to the profession; for we cannot go any deeper, or any farther towards solving the

---

*An address delivered at the 33rd annual Commencement of the Baltimore College of Dental Surgery, 1873.

"hidden problems" than our scientific knowledge will carry us; for all true scientific achievement, necessitates the application of exact learning.

If we wish to investigate the source of a river, we must seek its fountain head, "as we are not likely to see or comprehend the things of which we know nothing."

The power to seek information and the tact of knowing how to use it comprehensively, in relieving the ills of suffering humanity, and the advancement of science, is the goal to be reached only by laborious and continuous study; then, let us take no step backward, but keep up with the leaders of the profession.

We argue, not because we love our profession less, but because we love it more, and desire to arouse its individual members to be men of greater scholastic, and scientific attainments, raising individual standards higher, and thereby make it, what it should be, a peer of any the *learned* professions.

---

ARTICLE II.

